# Rate of atrial fibrillation and flutter induced tachycardiomyopathy in a cohort of hospitalized patients with heart failure and detection of indicators for improved diagnosis

**DOI:** 10.3389/fcvm.2022.940060

**Published:** 2023-01-12

**Authors:** Lynn Ermert, Fabienne Kreimer, Daniel R. Quast, Andreas Pflaumbaum, Andreas Mügge, Michael Gotzmann

**Affiliations:** ^1^University Hospital St. Josef-Hospital Bochum, Cardiology and Rhythmology, Ruhr-University Bochum, Bochum, Germany; ^2^University Hospital St. Josef-Hospital Bochum, Internal Medicine, Ruhr-University Bochum, Bochum, Germany

**Keywords:** tachycardiomyopathy, atrial fibrillation, atrial flutter, heart failure with reduced ejection fraction, diagnosis

## Abstract

**Background:**

Atrial fibrillation (AF) and atrial flutter (AFL) induced tachycardiomyopathy (TCM) has been known to cause reversible heart failure (HF) for many years. However, the prevalence of the disease is unknown, and diagnosis is challenging. Therefore, the aim of the present study was (1) to assess the rate of AF/AFL induced TCM and (2) to identify indicators for diagnosis.

**Methods:**

Consecutively, all patients with a diagnosis of HF who were hospitalized in our department within 12 months were reviewed. For the main analysis, all patients with HF with reduced ejection fraction (HFrEF) and AF or AFL were included. AF/AFL induced TCM was diagnosed when there was at least a 10% improvement in left ventricular ejection fraction under rhythm or rate control within 3 months. Patients with HFrEF with AF/AFL but without TCM served as control group.

**Results:**

A total of 480 patients were included. AF/AFL induced TCM occurred in 26 patients (5.4%) and HFrEF with AF/AFL in 53 patients (11%). Independent indicators of AF/AFL induced TCM were age<79 years [Odds ratio 5.887, confidence interval (CI) 1.999–17.339, *p* < 0.001], NT-pro-BNP <5,419 pg/mL (Odds ratio 2.327, CI 1.141–4.746, *p* = 0.004), and a resting heart rate >112 bpm (Odds ratio 2.503, CI 1.288–4.864, *p* = 0.001).

**Conclusion:**

Approximately 5% of all patients hospitalized for HF suffer from AF/AFL induced TCM. Improved discrimination of AF/AFL induced TCM to HFrEF with AF/AFL is possible considering age, NT-pro-BNP level, and resting heart rate >112 beats/minute. Based on these parameters, an earlier diagnosis and improved therapy might be possible.

## Introduction

Heart failure (HF) is a common cardiovascular disease with considerable impact on the prognosis of patients (1). Since many years it is recognized that a sustained high heart rate can be a reason or a contributing factor for the development of HF (2, 3). This phenomenon is therefore called tachycardiomyopathy (TCM). The underlying mechanisms and the pathophysiological principles have not been fully understood. Abnormalities in myocyte calcium levels, energy balance and subclinical cardiac ischemia have been described as contributing factors (2). In addition, there is evidence for changes in the morphology of cardiomyocytes and mitochondria, accompanied by macrophage-dominated cardiac inflammation in the TCM (4, 5).

Because of the high prevalence of atrial fibrillation (AF) and atrial flutter (AFL), these two arrhythmias are the most common causes of TCM by far (2, 3). Previous studies primarily investigated patients who underwent catheter ablation. In these studies, patients with AF/AFL induced TCM were comparatively younger and more likely to have persistent AF than control groups (6, 7). Of importance, effective therapy for AF/AFL induced TCM [i.e., rhythm control or rate control (8)] results in a significant improvement in HF and normalization of ejection fraction (6, 7). Considering the good prognosis of TCM in general, it seems essential to identify appropriate patients and perform rhythm or rate control. Previous studies investigated echocardiographic parameters (9–11), serial determinations of NT-pro BNP (12), or parameters of cardiac MRI examination (13) to identify TCM. However, there are no universal recommendations on how to differentiate AF/AFL induced TCM from HF with reduced ejection fraction (HFrEF). Given these difficulties in diagnosis, the exact prevalence of AF/AFL induced TCM is not known.

The aim of the present study is to assess the rate of AF/AFL induced TCM. For this purpose, we studied a non-selected cohort of consecutively hospitalized patients with HF over a period of 12 months. Furthermore, we analyzed the characteristics of patients with AF/AFL induced TCM and how they differ from patients with HFrEF. The objective was to identify useful indicators for the identification of patients who would benefit significantly from rhythm or rate normalization.

## Materials and methods

This study is a retrospective, monocentric analysis. Consecutively, all patients with the diagnosis of HF who were hospitalized in the Cardiology and Rhythmology department of the St. Josef-Hospital Bochum within 12 months from November 2020 to October 2021 were analyzed. For patients who were hospitalized for HF more than once during the specified study period, the first admission was used for the analysis. According to current European Society of Cardiology (ESC) guidelines, patients were divided into preserved, mildly reduced, and reduced ejection fraction groups based on their left ventricular ejection fraction (LVEF) (14). The study was approved by the local ethics committee of the Ruhr-University Bochum (Number 22-7531).

## Inclusion criteria and data acquisition

For the main analysis, all patients with HFrEF (LVEF ≤40%) and AF or AFL were included. Patients with an age <18 years, a NT-pro-BNP <125 ng/L, continuous pacemaker stimulation or with other bradycardic or tachycardic cardiac arrhythmias were excluded. Patients’ data was collected using the medical history and the medical documentation (including medication, laboratory results, ECG and echocardiography). The ECG and the laboratory results on admission were used for the analysis. The diagnosis of AF and AFL was made according to the ESC guidelines (15). The diagnosis of permanent AF was made when AF persisted for more than 12 months. This included patients in whom a failed attempt at rhythmization was attempted.

Transthoracic echocardiography was obtained according to the guidelines of the American Society of Echocardiography and European Association of Cardiovascular Imaging (16). LVEF was obtained with the Simpson biplane method. Treatment of AF and AFL was in accordance with current ESC recommendations and included rate control and rhythm control depending on individual patient characteristics and evaluation by the attending physicians (15). Methods of sinus rhythm recovery included catheter ablation and electrical cardioversion. Rhythm control after catheter ablation was verified by 24-h Holter ECG in either an inpatient or outpatient setting.

### Definition of study subgroups

Current guidelines define TCM as a reversible cause of LV-dysfunction due to tachycardia. As there is a lack of standardized diagnostic criteria, the following characteristics were chosen for the definition of TCM: For the purpose of the present study, AF/AFL induced TCM was defined as the presence of (1) AF or AFL, (2) a LVEF ≤40% and (3) improvement of LVEF by at least 10% under rhythm or rate control within the first 3 months.

HF with reduced ejection fraction with AF or AFL but without AF/AFL induced TCM was defined if the following criteria were present: (1) AF or AFL, (2) a LVEF of ≤40% and either (3.1) no improvement in LVEF by at least 10% under rhythm or rate control within the first 3 months or (3.2) history of previously documented reduced LVEF (≤40%) despite rhythm or rate control. The follow-up examinations were performed in an inpatient or outpatient setting partly in our hospital outpatient clinic or also with the outpatient cardiologist.

### Statistics

In the statistical analysis, we compared the characteristics of patients with AF/AFL induced TCM and HFrEF with AF/AFL. Numerical values are expressed as mean ± standard deviation. Continuous variables were compared between groups using an unpaired *t*-test (for normally distributed variables) or Mann–Whitney U test (for non-normally distributed variables). χ^2^ analysis was used to compare categoric variables. All variables in Tables 1, 2 were evaluated for an association with AF/AFL induced TCM in a univariate regression analysis. All variables with a significant association were entered in a multivariate regression analysis to identify independent indicators of AF/AFL induced TCM. Receiver operating characteristic curves (ROC) were generated to define cutoff values for independent indicators. Results are presented as Odds ratio. A *P*-value < 0.05 was considered significant. All probability values reported are 2-sided. The statistical software SPSS 26 was used for statistical analysis.

**TABLE 1:** Clinical characteristics of study patients (*n* = 79).

	AF/AFL induced TCM (*n* = 26)	HFrEF with AF/AFL (*n* = 53)	*P*-value
Age (years)	68 ± 10.1	80.8 ± 8.9	<0.001
Women, *n* (%)	11 (42)	24 (45)	0.802
NYHA functional class (I/II/III/IV)	4/8/11/3	1/16/24/12	0.103
**Cardiovascular risk factors**			
Hypertension, *n* (%)	22 (85)	43 (81)	0.830
Diabetes mellitus, *n* (%)	4 (15)	19 (36)	0.053
Dyslipidemia, *n* (%)	12 (46)	28 (53)	0.522
Current smoking, *n* (%)	6 (23)	5 (9)	0.098
**Medical history**			
Type of AF (paroxysmal/persistent/ permanent) (*n*)	2/19/2	7/20/26	0.001
Coronary artery disease, *n* (%)	4 (15)	29 (55)	0.001
Previous myocardial infarction, *n* (%)	2 (8)	12 (23)	0.088
Coronary artery bypass grafting, *n* (%)	1 (4)	7 (13)	0.179
Stroke/TIA, *n* (%)	5 (19)	7 (13)	0.509
Chronic obstructive lung disease, *n* (%)	2 (8)	14 (26)	0.047
Peripheral artery disease, *n* (%)	1 (4)	12 (23)	0.032
Pacemaker, *n* (%)	1 (4)	6 (11)	0.272
ICD/CRT, *n* (%)	0 (0)	6 (11)	0.074

TCM, tachycardiomyopathy; HFrEF, heart failure with reduced ejection fraction; AF/AFL, atrial fibrillation or atrial flutter; TIA, transient ischemic attack; ICD, implantable cardioverter defibrillator; CRT, cardiac resynchronization therapy.

**TABLE 2:** Electrocardiographic, echocardiographic, and laboratory parameters of study patients (*n* = 79).

	AF/AFL induced TCM (*n* = 26)	HFrEF with AF/AFL (*n* = 53)	*P*-value
**ECG parameter at admission**			
Atrial fibrillation/atrial flutter	22/4	49/4	0.278
Heart rate at rest (bpm)	126 ± 30	99 ± 27	0.001
QRS complex (ms)	102 ± 26	113 ± 27	0.403
Left bundle branch block, *n* (%)	3 (12)	8 (15)	0.848
**Echocardiographic parameters**			
Left atrial diameter (mm)	46.5 ± 7.6	46.3 ± 6.4	0.932
Left ventricular ejection fraction (%)	28.4 ± 8.1	30.3 ± 8.4	0.330
Left ventricular end-diastolic diameter (mm)	51.2 ± 8.2	52.5 ± 7.2	0.556
Left ventricular end-systolic diameter (mm)	44.4 ± 7.6	45.4 ± 7.2	0.636
Left ventricular mass (g)	263 ± 65	287 ± 92	0.369
Pulmonary artery pressure (mmHg)	27.5 ± 9.4	37.2 ± 16.5	0.125
Aortic stenosis (none/mild/moderate/severe), *n*	26/0/0/0	47/1/4/1	0.364
Aortic regurgitation (none/mild/moderate/severe), *n*	19/7/0/0	31/17/4/1	0.363
Mitral regurgitation (none/mild/moderate/severe), *n*	3/17/4/2	5/31/14/3	0.739
Tricuspid regurgitation (none/mild/moderate/severe), *n*	6/14/5/1	12/19/16/6	0.430
**Laboratory parameters**			
Hemoglobin (g/dL)	14.2 ± 1.7	12.5 ± 2.2	0.001
GFR (ml/min/1.73 m^2^)	70.2 ± 22.4	58 ± 24	0.034
C-reactive protein (mg/L)	10.5 ± 14	24.6 ± 40	0.087
NT-pro-BNP (pg/mL)	4,714 ± 3,871	9,610 ± 8,966	0.016
Hs Troponin T (pg/mL)	31.3 ± 28.5	67.9 ± 92.9	0.162

TCM, tachycardiomyopathy; HFrEF, heart failure with reduced ejection fraction; AF/AFL, atrial fibrillation and flutter; bpm, beats per minute; GFR, glomerular filtration rate using CKD-EPI equation; NT-pro-BNP, N terminal pro brain natriuretic peptide; Hs, high sensitive.

## Results

**FIGURE 1:**
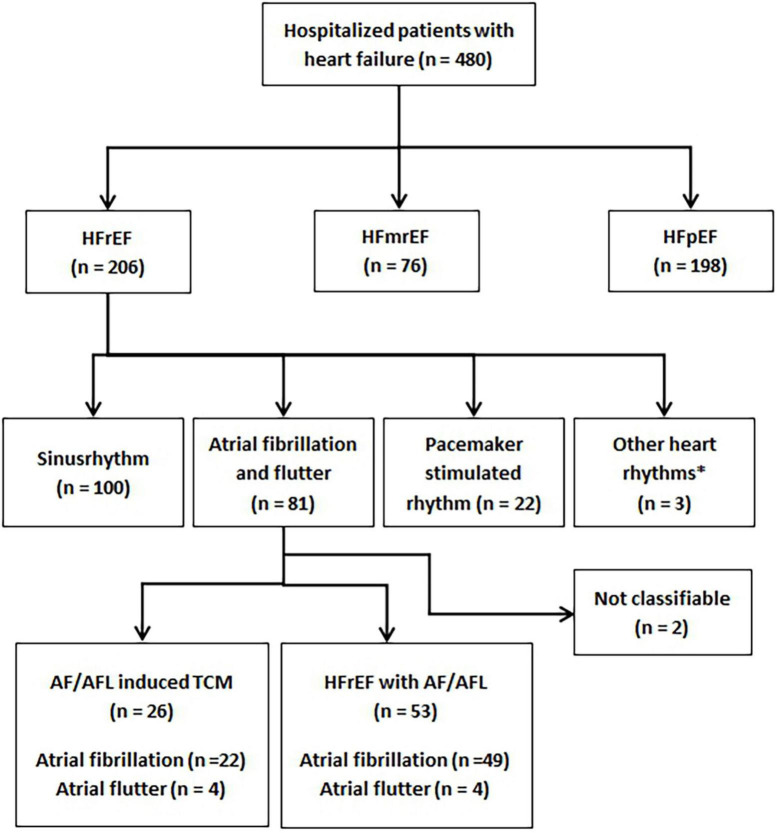
Flowchart demonstrating all patients diagnosed with heart failure during the study period and subgroups of patients. HFrEF, heart failure with reduced ejection fraction; HFmrEF, heart failure with mildly reduced ejection fraction; HFpEF, heart failure with preserved ejection fraction; TCM, tachycardiomyopathy; AF/AFL, atrial fibrillation or atrial flutter.

A total of 480 patients diagnosed with HF were treated at St. Josef Hospital during the study period. Mean age of patients was 76.2 ± 12.9 years, 222 patients (46%) were women. The median duration of hospitalization was 8.5 ± 7.4 days (range from 1 to 55 days). Intrahospital deaths occurred in 27 patients (5.6%). The distribution of patients according to the recommendations of the ESC guidelines is given in Figure 1.

### Subgroups

In total, 206 (42.9%) patients presented with HFrEF. Of these 206 patients, 100 (48.5%) were in sinus rhythm at the time of hospital admission, 22 (10.7%) had a pacemaker-stimulated rhythm, and three (1.5%) demonstrated another rhythm than AF or AFL (sustained ventricular tachycardia, *n* = 1; persistent AV block II°, *n* = 1; persistent AV block III°, *n* = 1). In the remaining 81 patients, classification was not possible in two patients (2.5%) because neither the history nor the follow-up results were conclusive. These two patients were excluded from the analysis.

Thus, 79 patients formed the final study cohort. The cardiac rhythm on admission was AFL in eight (10.1%) patients and AF in 71 (89.9%) patients (Table 2). According to their respective medical history, 28 (35.4%) patients had known permanent AF. There were 39 (49.4%) patients with persistent AF and nine (11.4%) patients with paroxysmal AF. Three (3.8%) patients had isolated AFL without history of AF.

Atrial fibrillation and flutter induced TCM was diagnosed in 26 (33%) patients and HFrEF with AF/AFL in 53 (67%) patients based on medical history and echocardiographic follow-up. The frequencies of the different patient subgroups in relation to the total number of patients with HF are presented in Figure 1.

### AF/AFL induced TCM versus HFrEF with AF/AFL

In the HFrEF with AF/AFL subgroup, permanent AF was present in 26 (49%) patients, contrasting only two (8%) patients with permanent AF in the subgroup with AF/AFL induced TCM (*p* = 0.001). Patients with HFrEF with AF/AFL were significantly older, had more frequently coronary artery disease and peripheral arterial disease than patients with AF/AFL induced TCM (Table 1). In addition to a significantly higher heart rate on admission, patients with AF/AFL induced TCM displayed several significant differences in laboratory parameters compared with patients with HFrEF with AF/ALF (Table 2).

In the group of HFrEF with AF/AFL, five (9.4%) patients died during hospitalization [one (20%) with acute cardiac decompensation due to acute myocardial infarction, four (80%) with prolonged cardiac decompensation]. In the group of patients with AF/AFL induced TCM, no patient died during hospitalization.

### Medical treatment and follow-up

In the subgroup with HFrEF with AF/AFL, primary rate-control therapy was performed in 35 (66%) patients. One patient converted spontaneously to sinus rhythm. In the remaining 17 patients, primary rhythm control was performed. This rhythm control also included several different therapies in individual patients: electrical cardioversion (*n* = 15), ablation of the cavotricuspid isthmus (*n* = 1), pulmonary vein isolation (*n* = 4), cardiac resynchronization therapy and AV node ablation (*n* = 2).

In the subgroup with AF/AFL induced TCM, rate-control therapy was primarily performed in five (19%) patients; one patient in this subgroup spontaneously converted to sinus rhythm.

In the remaining 21 patients, primary rhythm control was performed. In this subgroup, as well, several different therapies were performed in individual patients: electrical cardioversion (*n* = 19), ablation of the cavotricuspid isthmus (*n* = 5), pulmonary vein isolation (*n* = 12), cardiac resynchronization therapy and AV node ablation (*n* = 1).

A total of 14 (17.7%) patients received amiodarone therapy at discharge, and patients with AF/AFL induced TCM were significantly more likely to receive amiodarone than patients with HFrEF with AF/AFL.

There were no other notable differences between the subgroups in heart failure medication at discharge. Medications at discharge are listed in the table in the supplements (Supplementary Table 1).

At follow-up, surviving patients in the HFrEF with AF/AFL subgroup (*n* = 48) presented with sinus rhythm [*n* = 10 (21%)], AF [*n* = 36 (75%)] and pacemaker stimulated rhythm [*n* = 2 (4%)]. Mean heart rate at rest in the 3 month was 74.5 ± 8.9 beats per minute in AF/AFL induced TCM and 80 ± 12.8 beats per minute in HFrEF with AF/AFL (*p* = 0.069).

There was a significant (however, clinically minor) improvement in LVEF (31.6 ± 8.1% at baseline vs. 34.9 ± 11.8% at follow-up; *p* = 0.028).

In contrast, the AF/AFL induced TCM subgroup presented with sinus rhythm in 20 (77%) patients and AF in 6 (23%) patients. In AF/AFL induced TCM, a more pronounced (and clinically major) improvement in mean LVEF was observed (28.4 ± 8.1% at baseline vs. 49.4 ± 9.3% at follow-up; *p* < 0.001).

### Indicators of AF/AFL induced TCM

On univariate analysis, age, type of AF (paroxysmal/persistent/permanent), coronary artery disease, heart rate, hemoglobin, glomerular filtration rate and NT-pro-BNP were significantly related to AF/AFL induced TCM. Stepwise multivariable analysis identified age, heart rate and NT-pro-BNP as independent indicators of AF/AFL induced TCM.

**FIGURE 2:**
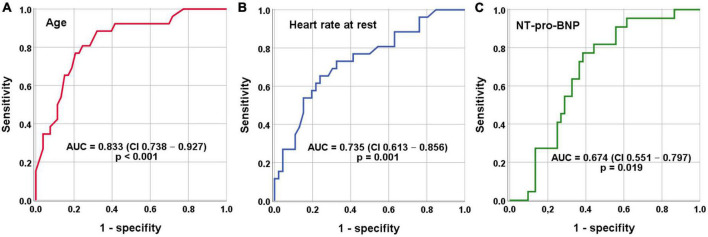
Receiver operating characteristics (ROC) analysis for indication of AF/AFL induced TCM. **(A)** Cut-off value age <79 years (sensitivity = 89% and specificity = 68%). **(B)** Cut-off value heart rate at rest >112 beats per minute (sensitivity = 73% and specificity = 67%). **(C)** Cut-off value NT-proBNP <5,419 pg/mL (sensitivity = 73% and specificity = 64%).

Using receiver operating characteristic (ROC) analysis cut-off values for independent indicators separating study patients were as follows: Age <79 years, Heart rate >112 beats per minute and NT-proBNP <5,419 pg/mL (Figure 2 and Table 3).

**TABLE 3:** Multivariate regression analysis for the indicators of AF/AFL induced TCM.

	Odds ratio	95% CI	*P*-value
Age < 79 years	5.887	1.999–17.339	<0.001
Heart rate at rest > 112 bpm	2.503	1.288–4.864	0.001
NT-pro-BNP < 5,419 pg/mL	2.327	1.141–4.746	0.004

TCM, tachycardiomyopathy; AF/AFL, atrial fibrillation and flutter; CI, confidence interval; NT-pro-BNP, N terminal pro brain natriuretic peptide.

## Discussion

The first main finding of this study is that AF/AFL induced TCM is present in one in 20 patients in an unselected cohort of patients hospitalized for HF. In the subgroup of patients with HFrEF, AF/AFL induced TCM is found in one in 8 patients (Figure 1). Given the relatively high prevalence of the disease and the therapeutic options available, the identification of AF/AFL induced TCM is imperative for clinicians. The second main finding of our study is that differentiation of AF/AFL induced TCM versus HFrEF with AF/AFL is feasible using age, resting heart rate, and NT-pro-BNP (Table 3 and Figure 2).

### Prevalence of AF/AFL induced TCM

Although TCM can occur in several tachycardic arrhythmias, AF and AFL are the most frequent underlying diseases of TCM (17). Therefore, previous studies have been conducted primarily in patient cohorts undergoing catheter ablation. In this particular collective, the prevalence of tachymyopathy was 8–14% (7, 18–20). In the subgroup of patients with LVEF <40%, the prevalence even exceeded 50% (7, 19).

Recently, in a prospective observational study, Stronati et al. (21) studied patients admitted for acute HFrEF who had evidence of atrial or ventricular arrhythmias on admission. Among patients diagnosed with TCM, AF (77.6%) and AFL (15%) were the most common underlying arrhythmias. In patients with diagnosis of heart failure on admission, the prevalence of TCM was approximately 9% (21).

The present study examined a non-selected collective of patients hospitalized for HF over a 12-month period. Thus, the present collective substantially differs from the collectives of other studies (7, 18–20). From a clinical point of view, the present study may therefore be more suitable to estimate the prevalence of AF/AFL induced TCM in relation to all patients with HF. Overall, AF/AFL induced TCM affected approximately 5% of all patients with a diagnosis of HF.

In our study, the group of patients with HFrEF was the largest subgroup (Figure 1). Stratification was performed based on heart rhythm on ECG on admission, and nearly 40% of these patients had AF or AFL. AF/AFL induced TCM occurred in more than 30% of these patients. Although the rates in our study are somewhat lower than in previous studies (21), our findings underline the clinical significance of the disease.

### Indicators of AF/AFL induced TCM

In contrast to many other diseases leading to heart failure, TCM is a principally reversible disease with a good prognosis when treated effectively (2, 3). Therefore, the diagnosis of a TCM is of utmost clinical importance. Despite the high relevance of the disease, clinicians may often struggle to identify TCM as there are currently no universal recommendations on how to distinguish TCM from other diseases. Several studies have approached this dilemma: Patients with TCM demonstrated a lower left ventricular end-diastolic diameter (LVEDD) than patients with dilated cardiomyopathy (9, 10). An LVEDD of ≤61 mm was able to distinguish TCM from dilated cardiomyopathy with a sensitivity of 100% and a specificity of 71% in one study (9). However, it remains unclear how this data translates to a heterogeneous study population without defined dilated cardiomyopathy. Contrasting previous study results, in the present cohort of unselected patients, echocardiographic parameters were not able to adequately distinguish patients with AF/AFL induced TCM from patients with HFrEF with AF/AFL (Table 2).

However, our study demonstrated that patients with AF/AFL induced TCM had lower NT-pro-BNP levels than patients with HFrEF and AF/AFL (Table 2). Moreover, NT-pro-BNP was an independent indicator of AF/AFL induced TCM (Table 3). These results suggest that the extent of myocardial injury in patients with AF/AFL induced TCM is less than that in patients with HFrEF with AF/AFL. This might also explain the better prognosis of AF/AFL induced TCM observed in other studies (4).

Previous studies demonstrated that TCM patients were comparatively younger than control groups (6, 7). The higher percentage of older patients with HFrEF with AF/AFL might be due to the greater proportion of comorbidities (coronary artery disease, peripheral arterial disease, renal failure, etc.) (Table 1). Age was associated with AF/AFL induced TCM in the present study as well (Table 1 and Figure 2). Moreover, we were able to identify a cutoff age of <79 years to discriminate patients with AF/AFL induced TCM and HFrEF with AF/AFL with good sensitivity and moderate specificity. The present results therefore extent the results of previous studies, giving clinicians a more detailed indication on when to suspect AF/AFL induced TCM. It should also be mentioned that older patients suffered from permanent AF to a higher extent, which also indicates more advanced and longer-lasting disease.

Interestingly, a definite cutoff for a heart rate that can induce TCM has not been defined, yet. For many years, a ventricular heart rate of >100 beats/minute has been suspected to be harmful (22). In contrast, the RACE (Race Control Efficacy in Permanent Atrial Fibrillation) II randomized controlled trial in patients with permanent AF found no difference in clinical events of rate-controlled therapy with a heart rate of <110 beats/minute compared with a heart rate of <80 beats/minute (23). This finding was also corroborated by an analysis of data from AFFIRM (Atrial Fibrillation Follow-up Investigation of Rhythm Management) (24). Therefore, current ESC guidelines recommend lenient rate control except for TCM (15). In our study, a higher resting heart rate on admission was associated with AF/AFL induced TCM (Figure 2). The optimal cut-off value for differentiating AF/AFL induced TCM from HFrEF with AF/AFL was >112 beats/minute (Figure 2). Our study thus suggests that this resting heart rate of 112 beats/minute should not be exceeded in the treatment of patients with AF and AFL.

### Limitations

Previous studies have defined TCM differently as there are no universally recognized diagnostic criteria or cut-off values. In some cases, patients with arrhythmias other than AF or AFL were included. In some cases, the study periods were longer and the improvement in LVEF had to be more marked. In the present study, a 10% improvement in LVEF within 3 months was used to define TCM. Therefore, a comparison of our results with those of other studies is only possible to a limited extent. Due to the retrospective design of the present study and since there were no universally accepted parameters to differentiate AF/AFL induced TCM from HFrEF with AF/AFL, an investigator bias cannot fully be excluded. While the present study was conducted over a period of 12 months, the number of patients with the diagnosis of AF/AFL induced TCM and the comparative collective remained relatively small. Strengths of the present study include the unselected cohort of patients, the clinical approach to the phenomenon of AF/AFL induced TCM and the clear identification of parameters to facilitate the discrimination of AF/AFL induced TCM and HFrEF with AF/AFL.

## Conclusion

Approximately 5% of all patients hospitalized for HF suffer from AF/AFL induced TCM, which in principle is a reversible condition. Improved discrimination of AF/AFL induced TCM to HFrEF with AF/AFL is possible considering age, NT-pro-BNP level, and resting heart rate >112 beats/minute. Based on these parameters, an earlier diagnosis might be possible, and a more effective therapy could be initiated.

## Data availability statement

The raw data supporting the conclusions of this article will be made available by the authors, without undue reservation.

## Ethics statement

The studies involving human participants were reviewed and approved by Ruhr-University Bochum (Number 22-7531). The patients/participants provided their written informed consent to participate in this study.

## Author contributions

MG, LE, and FK contributed to conception and design of the study. LE organized the database. MG performed the statistical analysis. MG and LE wrote the first draft of the manuscript. DQ, AM, and AP wrote sections of the manuscript. All authors contributed to manuscript revision, read, and approved the submitted version.
