# Supplementary material for: Rate of atrial fibrillation and flutter induced tachycardiomyopathy in a cohort of hospitalized patients with heart failure and detection of indicators for improved diagnosis
Source: Front Cardiovasc Med. 2023 Jan 12;9:940060. doi: 10.3389/fcvm.2022.940060 (PMC9878112; doi:10.3389/fcvm.2022.940060)
Supplement: Supplementary file 1 [file Table_1.DOCX]

**Supplementary Table 1:** Medication on discharge of surviving study patients on discharge (n = 74)

|  | AF/AFL induced TCM | HFrEF with AF/AFL | p value |
| --- | --- | --- | --- |
|  | (n = 26) | (n = 48) |  |
| ACE inhibitors, n (%) | 8 (32) | 22 (45) | 0.385 |
| Angiotensin-receptor blockers, n (%) | 7 (28) | 11 (22) | 0.506 |
| ARNI, n (%) | 6 (24) | 11 (22) | 0.778 |
| Beta blockers, n (%) | 25 (100) | 46 (94) | 0.077 |
| Thiazide diuretics, n (%) | 2 (8) | 6 (12) | 0.634 |
| Loop diuretics, n (%) | 17 (68) | 45 (92) | 0.054 |
| Aldosterone antagonists, n (%) | 14 (56) | 24 (49) | 0.418 |
| Amiodarone, n (%) | 9 (36) | 5 (10) | 0.005 |

TCM, tachycardiomyopathy; HFrEF, Heart failure with reduced ejection fraction; AF/AFL, atrial fibrillation and flutter; ACE, Angiotensin-converting-enzyme inhibitors; ARNI, Angiotensin-receptor blocker & neprilysin inhibitor;
